# Disrupted dynamic functional connectivity of hippocampal subregions mediated the slowed information processing speed in late-life depression

**DOI:** 10.1017/S0033291722003786

**Published:** 2023-10

**Authors:** Ben Chen, Mingfeng Yang, Xiaomei Zhong, Qiang Wang, Huarong Zhou, Meiling Liu, Min Zhang, Le Hou, Zhangying Wu, Si Zhang, Gaohong Lin, Yuping Ning

**Affiliations:** 1 Center for Geriatric Neuroscience, The Affiliated Brain Hospital of Guangzhou Medical University, Guangzhou, Guangdong Province, China; 2 The First School of Clinical Medicine, Southern Medical University, Guangzhou, Guangdong Province, China; 3 Department of Neurology, The Affiliated Brain Hospital of Guangzhou Medical University, Guangzhou, Guangdong Province, China; 4 Guangdong Engineering Technology Research Center for Translational Medicine of Mental Disorders, Guangzhou, China

**Keywords:** Cognitive impairment, dynamic functional connectivity, hippocampus, information processing speed, late-life depression, MRI

## Abstract

**Background:**

Slowed information processing speed (IPS) is the core contributor to cognitive impairment in patients with late-life depression (LLD). The hippocampus is an important link between depression and dementia, and it may be involved in IPS slowing in LLD. However, the relationship between a slowed IPS and the dynamic activity and connectivity of hippocampal subregions in patients with LLD remains unclear.

**Methods:**

One hundred thirty-four patients with LLD and 89 healthy controls were recruited. Sliding-window analysis was used to assess whole-brain dynamic functional connectivity (dFC), dynamic fractional amplitude of low-frequency fluctuations (dfALFF) and dynamic regional homogeneity (dReHo) for each hippocampal subregion seed.

**Results:**

Cognitive impairment (global cognition, verbal memory, language, visual–spatial skill, executive function and working memory) in patients with LLD was mediated by their slowed IPS. Compared with the controls, patients with LLD exhibited decreased dFC between various hippocampal subregions and the frontal cortex and decreased dReho in the left rostral hippocampus. Additionally, most of the dFCs were negatively associated with the severity of depressive symptoms and were positively associated with various domains of cognitive function. Moreover, the dFC between the left rostral hippocampus and middle frontal gyrus exhibited a partial mediation effect on the relationships between the scores of depressive symptoms and IPS.

**Conclusions:**

Patients with LLD exhibited decreased dFC between the hippocampus and frontal cortex, and the decreased dFC between the left rostral hippocampus and right middle frontal gyrus was involved in the underlying neural substrate of the slowed IPS.

## Introduction

Late-life depression (LLD), which affects approximately 3.5 to 7.5% of the geriatric population, is one of the most common psychiatric disorders and poses a great socioeconomic burden by increasing the costs of health care (Byers & Yaffe, 2011). Patients with LLD exhibited an overall cognitive impairment (e.g. information processing speed (IPS), executive function, memory, and language) (Linnemann & Lang, 2020), and their risk of developing dementia was 1.71–6.75 times higher than that of healthy elderly individuals (Kaup et al., 2016; Mirza et al., 2016). Among various cognitive deficits in patients with LLD, a slowed IPS appears to be the core domain, and it plays an essential role in downstream processes and other domains of cognitive function (Jungwirth et al., 2011; Nuño, GñomezBenito, Carmona, & Pino, 2021). Therefore, exploring the underlying neural substrate-downregulated IPS in LLD would provide a more in-depth understanding of their pathological mechanism and potential new targets for their early intervention. Previous studies have suggested that the slowed IPS was associated with disrupting reciprocal modulation between fronto-parietal, fronto-occipital, temporo-parietal and default modelling networks in health people (Silva et al., 2019, 2020). Interestingly, Wang et al., demonstrated that longitudinal changes in functional connectivity (FC) between the hippocampus and posterior cingulate cortex/precuneus were positively correlated with changes in IPS scores in patients with LLD (*n* = 14) (Wang et al., 2015), suggesting that functional abnormalities in the hippocampal subregion may be involved in the underlying pathological mechanism of slowed IPS in LLD.

The hippocampus not only plays a crucial role in spatial navigation and memory formation but is also an essential part of regulating the brain's response to psychosocial stress (Berger, Lee, Young, Aarsland, & Thuret, 2020) because hippocampal neurons express glucocorticoid receptors and the hippocampal inhibitory afferents suppress and regulate the release of hypothalamic corticotropin-releasing factor (Snyder, Soumier, Brewer, Pickel, & Cameron, 2011). It has been hypothesized that long-term exposure to stress or depression leads to an increase in circulating cortisol, acts on hippocampal glucocorticoid receptors and causes neurotoxicity, contributing to a smaller hippocampus and the development of dementia (Ouanes & Popp, 2019). Structural and functional abnormalities of the hippocampus have been repeatedly reported in patients with LLD (Kim & Han, 2021; Kim & Kim, 2021), but the results are not consistent, probably because most of these studies considered the hippocampus as a single homogeneous structure. Based on the cytoarchitectonic characteristics of the hippocampus, the hippocampus can be divided into the rostral and caudal subregions (Fan et al., 2016). The rostral hippocampus (most of the CA1 and subiculum) is preferentially connected to the orbitofrontal cortex and the amygdala, and is supposed to be involved in the regulation of memory and emotion, while the caudal hippocampus (most of the CA2-3 and dentate gyrus) is preferentially connected to the posterior parietal and retrosplenial cortices and is supposed to be involved in spatial processing (Zeidman & Maguire, 2016). Through investigations of hippocampal dysfunction at a subregional level, more details of structural and functional abnormalities have been found in patients with dementia (Kerchner et al., 2010) and depression (Shunkai et al., 2021). However, there was no evidence demonstrated the relationship between slowing IPS and hippocampus at a subregional level, and whether abnormalities of various hippocampal subregions may exhibit different effect on IPS in LLD patients remains unclear.

An increasing number of studies have reported that brain functional abnormalities can be evaluated more easily through dynamic analyses than through static measurements. When the dynamic sliding window method is used throughout the scanning procedure, the dynamic characteristics of brain function, such as the dynamic fractional amplitude of low-frequency fluctuations (dfALFF), dynamic regional homogeneity (dReHo) and dynamic functional connectivity (dFC), can be captured more effectively (Liao et al., 2019; Yan, Yang, Colcombe, Zuo, & Milham, 2017). Several researchers have successfully applied dynamic analyses to neuropsychiatric diseases, such as Alzheimer's disease (Cordova-Palomera et al., 2017), major depressive disorder (Luo et al., 2021), Parkinson's disease (Diez-Cirarda et al., 2017), bipolar disorder (Chen et al., 2022), and schizophrenia (Rashid, Damaraju, Pearlson, & Calhoun, 2014), which have provided a novel understanding of their pathophysiology. Additionally, abnormal dALFF and dFC were also shown in patients with major depressive disorders, and the decreased dFC between the left rostral hippocampus and left anterior lobe of the cerebellum was associated with working memory impairment in melancholic depression (Shunkai et al., 2021). However, no study has explored the dynamic functional characteristics in patients with LLD, and the relationship between dynamic functional abnormalities of hippocampal subregions and slowed IPS in LLD remains unclear.

Therefore, in the present study, we conducted a sliding window analysis to characterize the temporal variability in the spontaneous fluctuations of activity and connectivity of hippocampal subregions in patients with LLD in comparison with a group of healthy elderly people, and explored the relationships between dynamic functional abnormalities of the hippocampus and cognitive impairment in patients with LLD. We hypothesized that the IPS is mediated by dynamic dysfunction of the rostral hippocampus in patients with LLD.

## Method

### Participants

A total of 223 participants [134 patients with LLD and 89 healthy controls (HCs)] were recruited from the Affiliated Brain Hospital of Guangzhou Medical University and from the community of Guangzhou. All of the participants or their legal guardians provided written informed consent before taking part in our study. This study was approved by the Ethics Committee of The Affiliated Brain Hospital of Guangzhou Medical University (2014, 078).

The inclusion criteria for LLD patients were as follows: (1) were at least 55 years old and right-handed; (2) met the Diagnostic and Statistical Manual of Mental Disorders, Fourth Edition (DSM-IV), criteria for major depressive disorder after 55 years old; and (3) had their clinical stage and diagnosis confirmed by trained physicians at the hospital. HCs were included if they (1) were right-handed, (2) exhibited normal cognitive function, and (3) had no past history of depression.

The exclusion criteria of LLD and HCs were as follows: (1) had other major psychiatric illness (such as schizophrenia and bipolar disorders); (2) had a physical disease that may cause mental abnormalities (such as hypothyroidism and anaemia); (3) had a major neurological disease (such as Parkinson's disease and stroke); (4) had claustrophobia or metal implants that precluded magnetic resonance imaging (MRI) scans; and (5) currently or previously had psychotic symptoms. The diagnoses and assessments were made by a neuropsychologist and a geriatric psychiatrist.

### Clinical measurements

Demographic information (sex, age, and years of education) and clinical history (duration of illness and number of depressive episodes) of all subjects were collected at enrolment. The severity of depressive symptoms was assessed using the 17-item Hamilton Depression Rating Scale (HAMD-17). All the scale assessments were completed by two trained professional psychiatrists who passed a concordance assessment.

### Neuropsychological assessments

After undergoing standard clinical assessments, participants were interviewed by neuropsychologists to assess global cognitive function using the Mini-mental State Examination (MMSE), following a battery of neuropsychological tests to assess performance with respect to 5 cognitive domains: IPS [the symbol digit modality test (SDMT), Stroop Colour and Word Test part A (Stroop A), and Trail-Making Test part A (TMT A)]; memory [the auditory verbal learning test (AVLT) and working memory test (WMT)]; language [the Boston Naming Test (BNT) and verbal fluency test (VFT)]; executive function [Stroop Colour and Word Test C (Stroop C) and Trail-Making Test part B (TMT B)]; and visuospatial skills [Clock Drawing Test 4 (CDT4) and Rey-Osterrieth complex figure test (ROCF)].

### MRI data acquisition

Subjects underwent MRI scans after the neuropsychological assessments. A Philips 3.0 T MR system (Achieva, Netherlands) at the Affiliated Brain Hospital of Guangzhou Medical University was used to acquire the imaging data. For each participant, an anatomical image was obtained with a sagittal 3D gradient echo. Sagittal resting-state fMRI datasets of the whole brain were obtained in 8 min with a single-shot gradient echo-planar imaging pulse sequence. The resting-state fMRI scanning parameters were as follows: TE = 30 ms; TR = 2000 ms; flip angle (FA) = 90 degrees; numbers of slices = 33; slice thickness = 4 mm; matrix size = 64 × 64; and field of view (FOV) = 220 × 220 mm.

### Image preprocessing

Resting-state fMRI data preprocessing was carried out using the Data Processing Assistant for Resting-State 5.1 (DPARSF 5.1) (Yan, Wang, Zuo, & Zang, 2016). The first ten volumes were removed to preserve steady-state data only. The remaining images were corrected for timing differences and head motion. A record of the head motion was provided after realignment correction. Participants who had > 2 mm maximum displacement in any plane, 2° of angular motion and 0.2 mm in mean frame-wise displacement (FD) were excluded from further analysis. Then, the motion-corrected images were spatially normalized into a standard Montreal Neurological Institute (MNI) echo planar imaging (EPI) template and resliced to a voxel size of 3 × 3 × 3 mm^3^ resolution. The images were smoothed using a 6 mm full width at half maximum (FWHM) Gaussian kernel for dFC and fALFF calculations. The data with linear trends and nuisance signals, such as white matter signals and cerebrospinal fluid, were removed, and the Friston-24 parameters of head motion were regressed out from each time series. To minimize the influence of head motion, the mean FD of each participant was regressed out in group-level analysis. To reduce the effect of low-frequency drifts and high-frequency noise, a bandpass filter (0.01 Hz < f < 0.1 Hz) was applied for the analysis of dFC and dReho.

### Analyses of dFC, dfALFF and dReHo of hippocampal subregions

The temporal variability in the spontaneous fluctuations of activity was assessed by dfALFF and dReHo, and connectivity was assessed by dFC (Chen et al., 2022; Luo et al., 2021). The left and right caudal hippocampus and left and right rostral hippocampus were selected as the seeds for calculating the dFC, dfALFF and dReho variability according to the Brainnetome Atlas (Brainnetome Atlas Viewer 1.0; http://atlas.brainnetome.org/) (Fan et al., 2016). The Hamming window was used to slide the whole-brain BOLD signals. A sliding window size of 50 TR and a window step of 1 TR were selected to evaluate the whole-brain dFC, dfALFF and dReho variability. For each sliding window, correlation maps were produced by computing the temporal correlation coefficient between the truncated time series of the seeds and all the other voxels. Consequently, 181 sliding window correlation maps were obtained for each participant. The obtained correlation maps were then converted to *z* value maps using Fisher's *r*-to-*z* transformation to improve the normality of the correlation distribution. Subsequently, we calculated the standard deviation of the *z* value at each voxel to assess the dFC variability.

In each window length, for a given voxel, the time series was first converted to the frequency domain using fast Fourier transformation. The square root of the power spectrum was computed and then averaged across a predefined frequency interval (0.01–0.1 Hz). The average square root was considered to be the fALFF at the given voxel (Yang et al., 2007). Then, the standard deviation of the fALFF values across all 181 windows was calculated to quantitatively depict the temporal dynamic characteristics of fALFF. Subsequently, we applied *z* standardization. ReHo reflects the degree of local regional neural activity coherence. Briefly, it was calculated as Kendall's coefficient of concordance (or Kendall's W) of the time course of a given voxel with those of its nearest neighbours (26 voxels) (Zang, Jiang, Lu, He, & Tian, 2004). The same sliding window analyses were applied to calculate the dReho variability of each voxel. The dReHo map was smoothed with FWHM = 6 mm, and *z* standardization was applied. Finally, the left and right caudal hippocampus and left and right rostral hippocampus were selected as the seeds for extracting the mean *Z* scores of dfALFF and dReHo.

### Statistical analyses

Demographic and clinical data were analysed by using SPSS 25.0 (SPSS, Chicago, IL, USA). The differences in demographic information and neuropsychological scores between the LLD group and the HC group were analysed using a two-sample *t* test, and a chi-squared test was used to compare sex differences. The mediation analyses were performed to investigate the relationship between group differences in LLD and HC (independent variable) and various cognitive scores (dependent variable), and the SDMT score was considered a mediator, with age, sex and years of education as covariates. The mediation model was calculated in PROCESS 3.4, and the level of confidence for all confidence intervals in output was 95%, with 5000 bootstrap samples.

To examine the difference in dFC variability patterns between the LLD group and HC group, a two-sample *t* test was performed on the standard deviation at each voxel. The multiple comparisons of the two-sample *t* test were corrected by using Gaussian random field (GRF) theory (voxel *p* < 0.001, cluster *p* < 0.01). A two-sample *t* test was conducted on the mean *Z* score of dfALFF and dReho in the left and right caudal hippocampus and left and right rostral hippocampus. Age, sex and years of education were included as factors.

Partial correlations were used to investigate the correlation between the neuropsychological scores and the values of dFC, dfALFF and dReHo for each significant region, controlling variables included age, sex, and year of education. False Discovery Rate correction at *p* < 0.05 was used to correct the results of correlation analyses. Regression analyses were used to further determine which neuropsychological scores were most associated with the neuroimaging indicators, independent variable included neuropsychological scores which showed significant correlation with dFCs in correlation analyses, dependent variable were dFC values, covariates of all regression included age, sex, and year of education. Specifically, a stepwise analysis method was used in the multiple linear regression analysis, with *p*(entry) 0.05, *p*(removal) 0.10. The regression model begins with no variable, then tests each neuropsychological score as it is added to the model, and keeps those that are deemed most statistically significant—repeating the process until the results are optimal. The mediation analyses were performed to investigate the relationships between HAMD score (independent variable) and different cognitive scores (dependent variable), and the neuroimaging indicators were considered mediators, with age, sex and years of education serving as covariates.

### Validation analysis

Another 2 supplementary window lengths (30 TRs and 70 TRs) were applied to validate the main results of dfALFF, dReho and dFC with the window length of 50 TRs.

## Results

### Demographic, clinical and neuropsychological information

The demographic, clinical and neuropsychological information of the LLD group and the HC group is listed in Table 1. Two participants in the LLD group and a participant in the HC group were excluded from further analysis because they did not meet the criteria of head motion control. No significant difference was found in age or sex distribution between the LLD group and HC groups (*p* > 0.05), and the LLD group exhibited fewer years of education than the HC group (*p* < 0.05). For the comparison of cognitive scores, significant differences were found in all assessments between the LLD group and the HC group (*p* < 0.05). Additionally, there was no significant difference in FD among the two groups (LLD 0.052 ± 0.024, NC 0.055 ± 0.021, *t* = 0.794, *p* = 0.454).

**Table 1.** Demographic, clinical and neuropsychological information of the LLD group and HC group

	NC (*n* = 88)	LLD (*n* = 132)	*t*/χ^2^	*p*
Male (%)	22 (25%)	28 (21%)	0.43	0.51
Age	67.1 ± 6.1	67.6 ± 7.0	−0.58	0.558
Education (years)	11.6 ± 2.8	8.7 ± 4.1	5.84	<0.001
Depression				
HAMD	1.5 ± 1.9	9.0 ± 7.1	9.70	<0.001
Disease duration (years)	–	6.9 ± 8.5	–	–
Number of episodes	–	2.3 ± 2.6	–	–
Global cognition				
MMSE	27.4 ± 1.4	23.3 ± 4.9	7.59	<0.001
Information processing speed				
SDMT (s)	37.0 ± 9.0	26.0 ± 11.3	7.47	<0.001
Stroop A (s)	28.6 ± 7.1	35.5 ± 13.3	−4.09	<0.001
TMT A (s)	44.4 ± 12.8	64.9 ± 26.2	−6.24	<0.001
Memory				
AVLT N1-3	21.0 ± 4.7	16.3 ± 6.0	6.04	<0.001
AVLT N4	7.6 ± 1.8	5.1 ± 2.8	7.33	<0.001
AVLT N5	7.1 ± 2.1	4.3 ± 3.2	7.32	<0.001
AVLT N6	7.1 ± 1.9	4.1 ± 3.2	7.23	<0.001
WMT	4.2 ± 1.6	2.3 ± 1.6	7.16	<0.001
Executive function				
TMT B (s)	57.9 ± 17.4	87.0 ± 36.2	−6.50	<0.001
Stroop C (s)	77.3 ± 21.4	98.2 ± 37.9	−4.32	<0.001
Language				
BNT	23.5 ± 2.4	19.9 ± 3.5	7.69	<0.001
VFT	11.9 ± 4.0	8.8 ± 4.4	5.32	<0.001
Visuospatial skill				
CDT4	3.3 ± 0.9	4.0 ± 0.1	6.97	<0.001
ROCF	14.3 ± 5.6	8.3 ± 6.3	6.67	<0.001

MMSE, Mini-Mental State Examination; AVLT N1-3, Auditory Verbal Learning Test Immediate recall; AVLT N4, Auditory Verbal Learning Test Short-term delayed recall; AVLT N5, Auditory Verbal Learning Test Long-term delayed recall; AVLT N6, Auditory Verbal Learning Test Recognition; TMT, Trail-Making Test; Stroop, The Stroop Colour and Word Test; BNT, Boston Naming Test; VFT, Verbal Fluency Test; SDMT, Symbol-Digit Modalities Test; WMT, Working Memory Test. ROCF, Rey-Osterrieth Complex; CDT, Clock Drawing Task.

### Mediated effect of slowed IPS on cognitive impairment

**Fig. 1.**
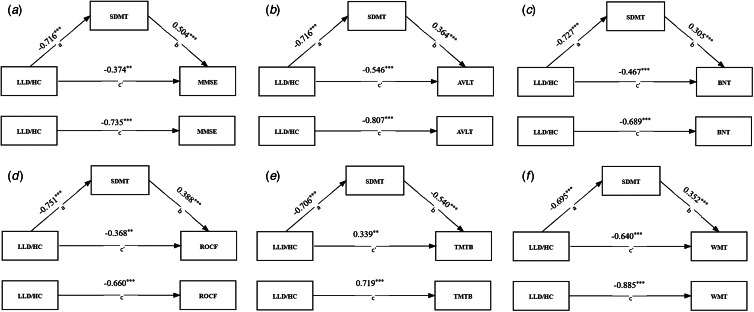
The mediated effect of a slowed IPS on cognitive impairment in patients with LLD. a. The SDMT exhibited a partially mediated effect on the group difference (LLD/HC) of MMSE; b. The SDMT exhibited a partially mediated effect on the group difference (LLD/HC) of AVLT; c. The SDMT exhibited a partially mediated effect on the group difference (LLD/HC) of BNT; d. The SDMT exhibited a partially mediated effect on the group difference (LLD/HC) of ROCF; e. The SDMT exhibited a partially mediated effect on the group difference (LLD/HC) of TMTB; f. The SDMT exhibited a partially mediated effect on the group difference (LLD/HC) of WMT. a: The effect of the independent variable on the mediating variable. b: The effect of the mediating variable on the dependent variable. c: The total effect of the independent variable on the dependent variable. c`: The direct effect of the independent variable on the dependent variable. SDMT: score of Symbol-Digit Modality Test. MMSE: Mini-mental State Examination. AVLT: Auditory Verbal Learning Test. WMT: Working Memory Test. BNT: Boston Naming Test. TMTB: Trail-Making Test B. ROCF: Rey-Osterrieth complex figure test. *: *p* < 0.05, **: *p* < 0.01, ***: *p* < 0.001.

According to the mediation analyses, the SDMT exhibited a partially mediated effect on the group difference (LLD/HC) of MMSE, AVLT, BNT, ROCF, TMTB and WMT (Fig. 1, and the statistical details are shown in the online Supplemental material), suggesting that the cognitive impairment (global cognition, verbal memory, language, visual–spatial skill, executive function and working memory) in patients with LLD was mediated by their slowed IPS.

### Comparison of the static fALFF, ReHo and FC of hippocampal subregions

**Fig. 2.**
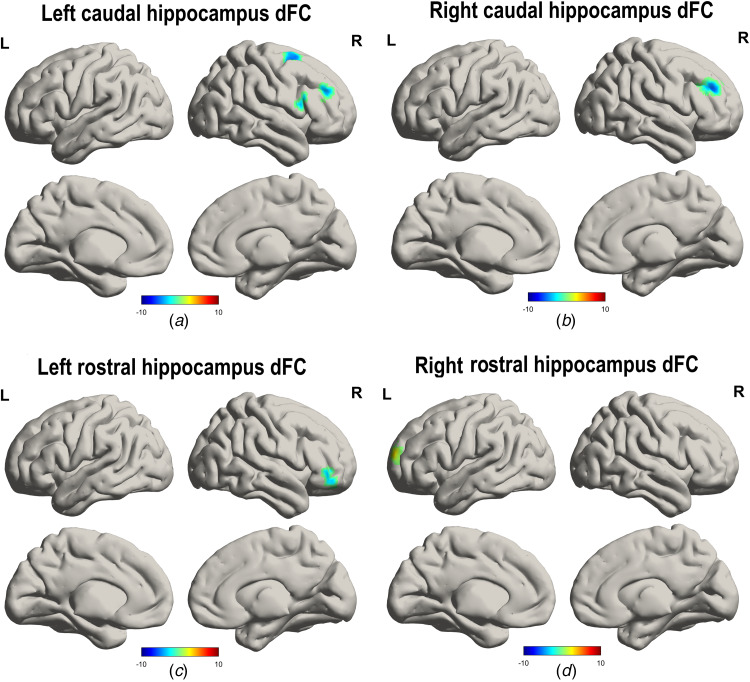
Comparison of the dFC of hippocampal subregions between the LLD group and the HC group. The LLD group exhibited decreased dFC between the a. left caudal hippocampus and right inferior frontal gyrus (opercular), left caudal hippocampus and right inferior frontal gyrus (triangular), left caudal hippocampus and right superior frontal gyrus; b. right caudal hippocampus and right middle frontal gyrus; c. left rostral hippocampus and right middle frontal gyrus (orbital); and d. the increased dFC between the right rostral hippocampus and left superior frontal gyrus (Table 2, Fig. 2d). LLD, late-life depression; HC, healthy control; dFC: dynamic functional connectivity. L, left; R, right.

There were no significant differences in static fALFF, Reho and FC hippocampal subregions between the LLD group and the HC group (S.Figure 1–2).

### Comparison of dFC, dfALFF and dReHo of hippocampal subregions

Compared with the HC group, the LLD group exhibited decreased dFC between the left caudal hippocampus and right inferior frontal gyrus (opercular), left caudal hippocampus and right inferior frontal gyrus (triangular), left caudal hippocampus and right superior frontal gyrus (Table 2, Fig. 2a), right caudal hippocampus and right middle frontal gyrus (Table 2, Fig. 2b), left rostral hippocampus and right middle frontal gyrus (orbital) (Table 2, Fig. 2c), and increased dFC between the right rostral hippocampus and left superior frontal gyrus (Table 2, Fig. 2d).

**Table 2.** Comparison of the dFC of hippocampal subregions between the LLD group and the HC group

Brain Regions	Peak MNI			Cluster size	Peak Intensity
	*x*	*y*	*z*		
Left caudal hippocampus dFC					
Right inferior frontal gyrus (opercular)	51	12	15	35	4.23
Right inferior frontal gyrus (triangular)	45	36	24	46	4.49
Right superior frontal gyrus	27	9	63	74	5.48
Right caudal hippocampus dFC					
Right middle frontal gyrus	42	39	27	117	5.85
Left rostral hippocampus dFC					
Right middle frontal gyrus (orbital)	33	45	−9	57	4.38
Right rostral hippocampus dFC					
Left superior frontal gyrus	−18	57	18	56	4.68

dFC, dynamic functional connectivity; MNI, Montreal Neurological Institute coordinates.

**Fig. 3.**
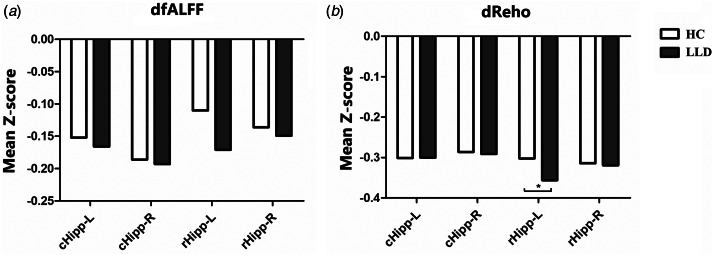
Comparison of the dfALFF and dReHo of hippocampal subregions between the LLD group and the HC group. The LLD group exhibited a lower dReho in the left rostral hippocampus (*t* = 2.10, *p* = 0.023). dfALFF, dynamic dynamic fractional amplitude of low-frequency fluctuations; dReho, dynamic regional homogeneity; cHipp, caudal hippocampus; rHipp, rostral hippocampus; L, left; R, right.

Compared with the HC group, the LLD group presented lower dReho in the left rostral hippocampus (*t* = 2.10, *p* = 0.023). There were no other significant differences in dfALFF or dReHo of hippocampal subregions between the LLD group and the HC group (Fig. 3).

### Correlation analyses

The dFC of various hippocampal subregions was associated with different neuropsychological scores. (1) The dFC between the left caudal hippocampus and right inferior frontal gyrus (opercular) was associated with VFT (*r* = 0.187, *p* = 0.028, corrected *p* = 0.068), Stroop A (*r* = −0.273, *p* = 0.001, corrected *p* = 0.020), WMT (*r* = 0.213, *p* = 0.012, corrected *p* = 0.043), and HAMD (*r* = −0.271, *p* = 0.001, corrected *p* = 0.020). (2) The dFC between the left caudal hippocampus and right inferior frontal gyrus (triangular) was associated with SDMT (*r* = 0.261, *p* = 0.002, corrected *p* = 0.020), Stroop A (*r* = −0.233, *p* = 0.006, corrected *p* = 0.030), WMT (*r* = 0.206, *p* = 0.015, corrected *p* = 0.047) and HAMD (*r* = −0.186, *p* = 0.029, corrected *p* = 0.068). (3) The dFC between the left caudal hippocampus and right superior frontal gyrus was associated with AVLT N1-3 (*r* = 0.139, *p* = 0.044, corrected *p* = 0.094), VFT (*r* = 0.205, *p* = 0.016, corrected *p* = 0.047), and WMT (*r* = 0.189, *p* = 0.026, corrected *p* = 0.066). (4) The dFC between the right caudal hippocampus and right middle frontal gyrus was associated with MMSE (*r* = 0.222, *p* = 0.009, corrected *p* = 0.037), AVLT N1-3 (*r* = 0.184, *p* = 0.008, corrected *p* = 0.036), BNT (*r* = 0.233, *p* = 0.006, corrected *p* = 0.030), SDMT (*r* = 0.258, *p* = 0.002, corrected *p* = 0.020), VFT (*r* = 0.206, *p* = 0.015, corrected *p* = 0.047), TMTB (*r* = −0.233, *p* = 0.006, corrected *p* = 0.030), ROCF (*r* = 0.237, *p* = 0.005, corrected *p* = 0.029), AVLT N5 (*r* = 0.222, *p* = 0.009, corrected *p* = 0.037), Stroop A (*r* = −0.221, *p* = 0.009, corrected *p* = 0.037), Stroop C (*r* = −0.185, *p* = 0.030, corrected *p* = 0.067), WMT (*r* = 0.246, *p* = 0.004, corrected *p* = 0.028), and HAMD (*r* = −0.293, *p* < 0.001, corrected *p* = 0.022). (5) The dFC between the left rostral hippocampus and right middle frontal gyrus (orbital) was associated with MMSE (*r* = 0.256, *p* = 0.002, corrected *p*= 0.020), AVLT N1-3 (*r* = 0.208, *p* = 0.002, corrected *p* = 0.020), BNT (*r* = 0.241, *p* = 0.004, corrected *p* = 0.028), SDMT (*r* = 0.329, *p* < 0.001, corrected *p* = 0.007), VFT (*r* = 0.285, *p* = 0.001, corrected *p* = 0.020), TMTB (*r* = −0.186, *p* = 0.029, corrected *p* = 0.068), ROCF (*r* = 0.263, *p* = 0.002, corrected *p* = 0.020), AVLT N4 (*r* = 0.214, *p* = 0.012, corrected *p* = 0.043), AVLT N5 (*r* = 0.192, *p* = 0.024, corrected *p* = 0.064), Stroop A (*r* = −0.243, *p* = 0.004, corrected *p* = 0.028), Stroop C (*r* = −0.285, *p* = 0.001, corrected *p* = 0.020), WMT (*r* = 0.200, *p* = 0.019, corrected *p* = 0.053), and HAMD (*r* = −0.189, *p* = 0.026, corrected *p* = 0.066). (6) The dFC between the right rostral hippocampus and left superior frontal gyrus was associated with HAMD (*r* = 0.237, *p* = 0.005, corrected *p* = 0.029).

### Regression analyses

**Fig. 4.**
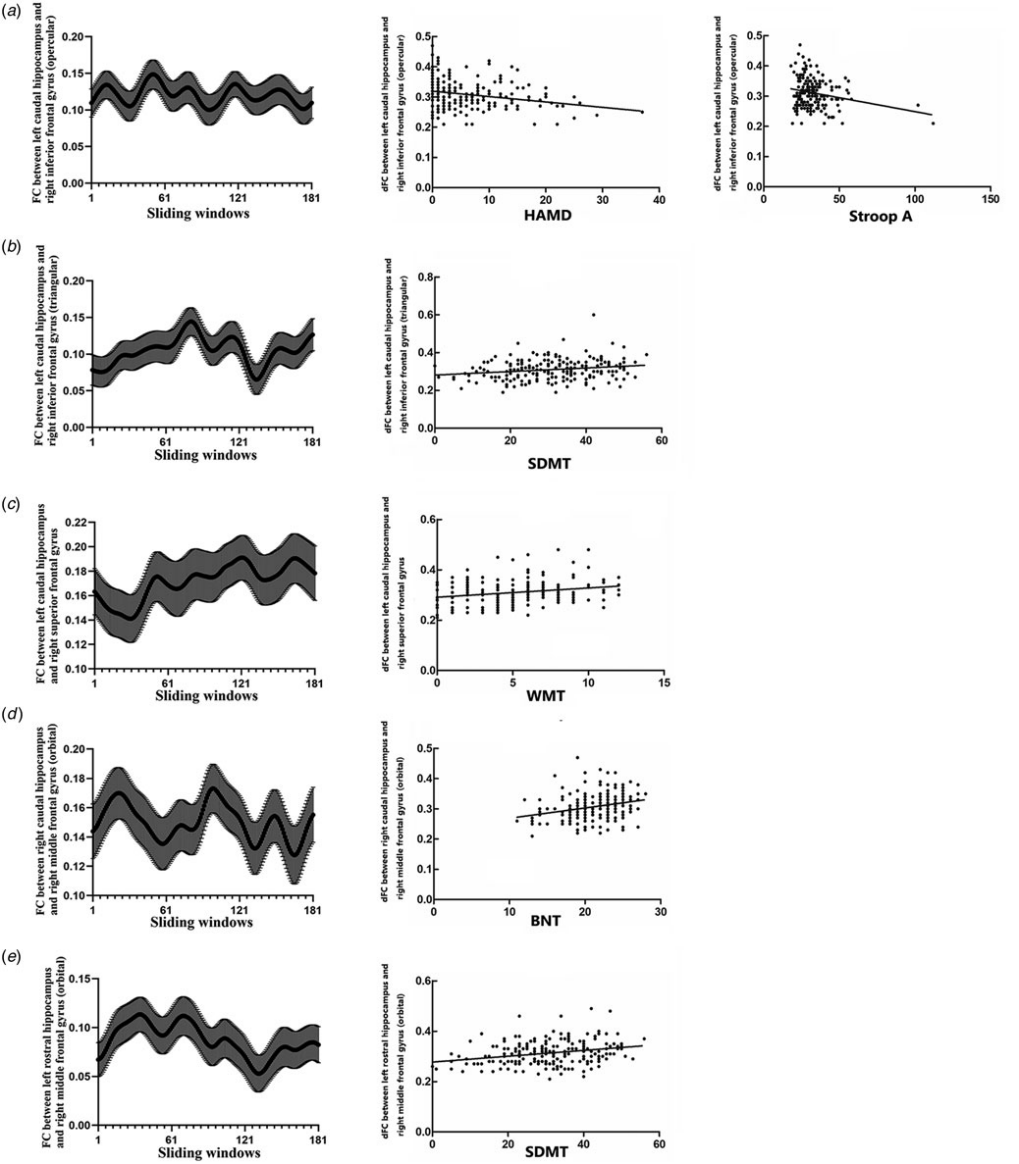
Association between the hippocampal dFC and neuropsychological variables. a, The dFC between the left caudal hippocampus and inferior frontal gyrus (opercular) was negatively associated with HAMD and Stroop A. b. The dFC between the left caudal hippocampus and inferior frontal gyrus (triangular) was most associated with SDMT. c. The dFC between the left caudal hippocampus and superior frontal gyrus was most associated with WMT. d. The dFC between the right caudal hippocampus and middle frontal gyrus was most associated with BNT. e. The dFC between the left rostral hippocampus and middle frontal gyrus (orbital) was most associated with SDMT.

The dFC between the left caudal hippocampus and right inferior frontal gyrus (opercular) was most strongly associated with HAMD (Table 3, Fig. 4a) and Stroop A (Table 3, Fig. 4b). The left caudal hippocampus and right inferior frontal gyrus (triangular) were most strongly associated with SDMT (Table 3, Fig. 4c), and the left caudal hippocampus and right superior frontal gyrus were most strongly associated with WMT (Table 3, Fig. 4d). The right caudal hippocampus and right middle frontal gyrus were most strongly associated with BNT (Table 3, Fig. 4e), and the left rostral hippocampus and right middle frontal gyrus (orbital) were most strongly associated with SDMT (Table 3, Fig. 4f).

**Table 3.** Regression analyses of the hippocampal dFC and neuropsychological variables

	*R*^2^	Variables	*β*	*t*	*p*
dFC between left caudal hippocampus and right inferior frontal gyrus (opercular)	0.112	HAMD	−0.241	−3.085	0.002
		Stroop A	−0.196	−2.504	0.013
dFC between left caudal hippocampus and right inferior frontal gyrus (triangular)	0.058	SDMT	0.240	2.934	0.004
dFC between left caudal hippocampus and right superior frontal gyrus	0.056	WMT	0.236	2.883	0.005
dFC between right caudal hippocampus and right middle frontal gyrus (orbital)	0.063	BNT	0.251	3.062	0.003
dFC between left rostral hippocampus and right middle frontal gyrus (orbital)	0.090	SDMT	0.300	3.714	<0.001

dFC, dynamic functional connectivity; SDMT, Symbol-Digit Modality Test; Stroop A, Stroop Colour and Word Test part A; WMT, Working Memory test; BNT, Boston Naming Test; HAMD, Hamilton Depression Rating Scale.

### Mediating effect of dynamic functional abnormalities on the relationship between depression and cognition

**Fig. 5.**
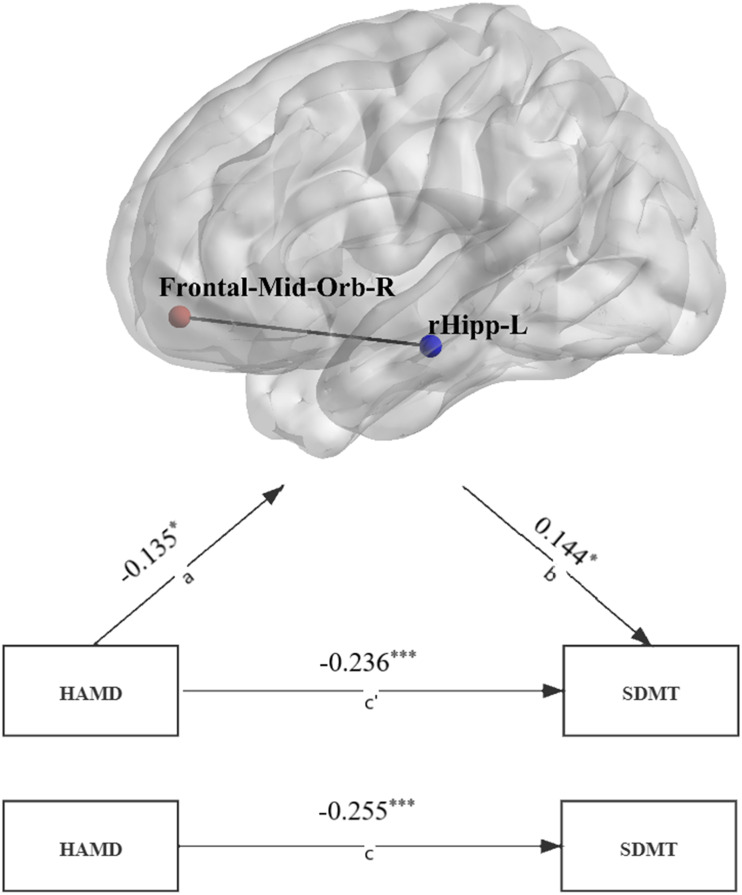
The association between depression and cognitive function was mediated by hippocampal dFC. a: The effect of HAMD scores on the dFC between the left rostral hippocampus and middle frontal gyrus (orbital). b: The effect of dFC between the left rostral hippocampus and middle frontal gyrus (orbital) on SDMT. c`: The direct effect of HAMD scores on the SDMT. c: The total effect of HAMD scores on the SDMT. dFC, dynamic functional connectivity; Frontal-Mid-Orb-R, right middle frontal gyrus (orbital); rHipp-L, left rostral hippocampus; HAMD, score of the 17-item Hamilton Depression Rating Scale; SDMT, score of the Symbol-Digit Modality Test. *: *p* < 0.05. ***: *p* < 0.001.

The total effect of HAMD scores on the SDMT was *β* = −0.255 (*p* < 0.001), and the indirect effect of HAMD scores on the SDMT mediated by the dFC between the left rostral hippocampus and right middle frontal gyrus (orbital) was *β* = −0.019 (BootLLCI = −0.049，BootULCI = −0.0003, Fig. 5). Furthermore, the remaining direct effect of HAMD scores on the SDMT was *β* = −0.236 (*p* < 0.001), with the effect of HAMD scores on dFC between the left rostral hippocampus and middle frontal gyrus (orbital) being *β* = −0.135 (*p* = 0.0498) and the effect of dFC between the left rostral hippocampus and right middle frontal gyrus (orbital) on SDMT being *β* = 0.144 (*p* = 0.017). In summary, the results above revealed that the dFC between the left rostral hippocampus and right middle frontal gyrus (orbital) was a mediator of the relationship between HAMD scores and SDMT. No significant mediated effect was found in dFC values on the association between the HAMD scores and the other neuropsychological variables.

## Validation results

The main results of 30 TRs and 70 TRs sliding-window length validated the main results (50 TRs) (S.Table 1–2, S.Figure 3–5 in the Supplementary Material).

## Discussion

The present study first explored the relationships between dynamic functional abnormalities of hippocampal subregions, cognitive impairment and depressive symptoms in patients with LLD, and the following results emerged. **First**, the slowed IPS mediated all domains of cognitive impairment in patients with LLD. **Second**, compared with the controls, patients with LLD exhibited abnormal dFC between all hippocampal subregions and the frontal cortex and decreased dReho in the left rostral hippocampus. **Third**, most hippocampal subregional dFCs (except dFC between the right rostral hippocampus and superior frontal gyrus) were negatively associated with the severity of depressive symptoms and positively associated with various domains of cognitive impairment. **Fourth**, the decreased dFC between the left rostral hippocampus and right middle frontal gyrus was a mediator of the relationship between the more severe depressive symptoms and the slowed IPS.

Currently, the processing speed hypothesis is one of the main neuropsychological hypotheses concerning the mechanism underlying cognitive impairment in LLD (Nuño et al., 2021). The processing speed hypothesis proposes that a slowing of IPS would have a negative impact on the higher cognitive functions of people with depression, and this has been proven by several studies (Hartog, Derix, Bemmel, Kremer, & Jolles, 2003; Jungwirth et al., 2011; Nebes, Butters, Mulsant, Pollock, & Reynolds, 2000). Consistently, the present results suggested that the slowed IPS partially mediated the impairment of global cognition, verbal memory, language, visuospatial skill, executive function and working memory in patients with LLD, suggesting that the slowed IPS contributes to the impairment in other cognitive domains. Furthermore, a previous study demonstrated that a slowed IPS predicted the severity of depressive symptoms at the one-year follow-up (Wu et al., 2021) and impaired the expectancies of the effect of antidepressants in LLD patients, which may help explain the lower antidepressant response among older adults (Rutherford, Choi, Choi, Mass, & Roose, 2021). Therefore, more attention should be given to the early intervention of slowed IPS in patients with LLD, and longitudinal studies are needed to further explore the relationship between IPS and other cognitive function in patients with LLD.

Both the hippocampus and the frontal cortex are important parts of the prefrontal–limbic network, which is now recognized as a key regulatory system involved in depression (Bennett, 2011). Previous studies have demonstrated disrupted static FC between the hippocampus and frontal cortex in patients with LLD (Wang et al., 2015), and the present study suggested that they were suffering abnormal temporal variability in the spontaneous fluctuations of activity and connectivity in hippocampal subregions, especially their dFC with the frontal cortex. Additionally, the abnormal dFCs between hippocampal subregions and the frontal cortex were associated with the severity of depressive symptoms and cognitive impairment. A possible explanation was that the abnormal dFCs between the hippocampal and frontal cortices may indicate that the ability to combine specialized information from distributed regions was impaired, leading to reduced efficiency in the functional connectivity of communication within the prefrontal–limbic network (Hutchison, Womelsdorf, Allen, Bandettini, & Corbetta, 2013). The disrupted prefrontal–limbic network may cause abnormal interaction with the basal ganglia, resulting in impaired goal-directed behaviour associated with the capacity to exclude negative thoughts, the attainment of rewards and the avoidance of punishments (Balleine & O Doherty, 2010), leading to depressive symptoms and cognitive impairment in patients with LLD.

In particular, the dFC between the left rostral hippocampus and middle frontal gyrus was a mediator of the relationship between the severity of depressive symptoms and IPS, which means that the exacerbation of depressive symptoms may slow the IPS by interrupting the functional communication between the rostral hippocampus and middle frontal gyrus. This mediated effect could be explained by the endocrine vicious cycle of LLD: the hippocampus inhibits the hypothalamo-pituitary-adrenal axis, and cortisol can be promoted by mineralocorticoid receptors and inhibited by glucocorticoid receptor activation of pyramidal cells in the hippocampal CA1 (rostral part of the hippocampus) (Kloet, Meijer, Nicola, Rijk, & Jols, 2018). The exacerbation of stress or depression elevates the level of cortisol, which can lead to hippocampal dysfunction and release of the inhibition of the hypothalamo-pituitary-adrenal axis, causing increased cortisol and inducing further hippocampal dysfunction (Linnemann & Lang, 2020). Because the middle frontal gyrus plays a critical role in regulating the interaction between dorsal and ventral attention networks (Penghui, Hua, Chunyan, & Qing, 2019), rostral hippocampal dysfunction may reduce its efficiency of functional integration with the middle frontal gyrus, disrupt the attention network and slow the IPS in patients with LLD. These results support that the middle frontal gyrus may serve as a potential target of neuromodulation for regulating the attention network (Penghui et al., 2019), suggesting that modulating the middle frontal gyrus may be a future direction for improving hippocampal dysfunction and slowing IPS and depressive symptoms in LLD patients. Longitudinal studies are needed to confirm the dynamic relationships between IPS, depressive symptoms and dFC between the rostral hippocampus and middle frontal gyrus, and task-fMRI studies are needed to provide a more in-depth understanding of how the IPS is influenced by the interaction of the rostral hippocampus and middle frontal gyrus.

It is well known that the rostral hippocampus is principally involved in the regulation of emotion and memory, while the caudal hippocampus is more associated with spatial processing. Interestingly, the present results suggested that the dFC of both the rostral and caudal hippocampus was associated with depression and cognitive impairment in patients with LLD, suggesting that the functions of different hippocampal subregions partially overlap. Additionally, these abnormal dFCs were not only associated with a slowed IPS but were also associated with impairment of global cognition, verbal memory, language, visuospatial skill, executive function and working memory in patients with LLD. The present results suggested that the reduced efficiency of the functional connectivity between the hippocampus and frontal cortex was widely involved in different cognitive processes, although the mediated effect was not as significant as that on IPS.

### Limitations

The present findings should be interpreted in light of several limitations. **First**, the present study is based on a cross-sectional design, and the relationships between cognitive impairment, depression and hippocampal dysfunction need to be further explored by a follow-up study. **Second**, the dFC reflects the correlation between the activity of the hippocampus and the frontal cortex, and further analyses via Granger causality or dynamic causal modelling could clarify how these regions interact with each other. Additionally, task-fMRI applying an SDMT design (Silva et al., 2019) could provide a more in-depth understanding of how hippocampal dFC is involved in the slowed IPS in patients with LLD. **Third**, the present study did not include biomarkers related to neuroplasticity inflammation and cortisol, and the underlying mechanism of hippocampal dFC mediating the relationship between depressive symptoms and cognitive impairment in LLD remains unclear. **Fourth,** previous studies suggested that motor or visual/auditory deficit play a role in the slowing IPS, and sensorimotor systems dysfunction has been found in LLD patients. Therefore, the present study excluded their potential effect by a qualitative way, that experienced neurologists have conducted systematic examinations for all subjects and excluded those with obvious motor or visual/auditory deficit. Future studies applying quantitative measurement for motor function and visual/ auditory ability could better clarify the relationship between hippocampal dFC and IPS in patients with LLD.

## Conclusion

Patients with LLD exhibited abnormal dFC between the hippocampus and frontal cortex, and the decreased dFC variability between the left rostral hippocampus and middle frontal gyrus was involved in the underlying neural substrate of the slowed IPS. Mapping the abnormalities of hippocampal subregions in patients with LLD provides a more in-depth understanding of their pathological mechanism and may reveal a potential direction for their interventions.

## Data Availability

The data that support the findings of this study are available from the corresponding author upon reasonable request.

## Supplementary material

For supplementary material accompanying this paper visit https://doi.org/10.1017/S0033291722003786. click here to view supplementary material
