# Supplementary material for: Disrupted dynamic functional connectivity of hippocampal subregions mediated the slowed information processing speed in late-life depression
Source: Psychol Med. 2023 Feb 20;53(14):6500–10. doi: 10.1017/S0033291722003786 (PMC10600940; doi:10.1017/S0033291722003786)
Supplement: Supplementary file 1 [file S0033291722003786sup001.docx]

**Mediated effect of slowed IPS on cognitive impairment**

The total effect of LLD/HC on the MMSE was β= -0.735 (P<0.001), the indirect effect of LLD/HC on the MMSE mediated by the SDMT was β= -0.361 (BootLLCI= -0.507，BootULCI=-0.231). Furthermore, the remaining direct effect of LLD/HC on the MMSE was β= -0.374 (P P=0.003), with the effect of LLD/HC on the SDMT being β= -0.716 (P<0.001) and SDMT on MMSE being β=0.504 (P<0.001) **(Figure 1B)**.

The total effect of LLD/HC on the AVLT was β= -0.807 (P<0.001), the indirect effect of LLD/HC on the SDMT mediated by the AVLT was β= -0.261 (BootLLCI= -0.404，BootULCI=-0.135). Furthermore, the remaining direct effect of LLD/HC on the AVLT was β= -0.546 (P P<0.001), with the effect of LLD/HC on the SDMT being β= -0.716 (P<0.001) and SDMT on AVLT being β=0.364 (P<0.001) **(Figure 1C)**.

The total effect of LLD/HC on the BNT was β= -0.689 (P<0.001), the indirect effect of LLD/HC on the BNT mediated by SDMT the was β= -0.221 (BootLLCI= -0.357，BootULCI=-0.111). Furthermore, the remaining direct effect of LLD/HC on the BNT was β= -0.467 (P P<0.001), with the effect of LLD/HC on the SDMT being β= -0.727 (P<0.001) and SDMT on BNT being β=0.305 (P<0.001) **(Figure 1D)**.

The total effect of LLD/HC on the ROCF was β= -0.660 (P<0.001), the indirect effect of LLD/HC on the ROCF mediated by the SDMT was β= -0.292 (BootLLCI= -0.445，BootULCI=-0.157). Furthermore, the remaining direct effect of LLD/HC on the ROCF was β= -0.368 (P P=0.012), with the effect of LLD/HC on the SDMT being β= -0.751 (P<0.001) and SDMT on ROCF being β=0.388 (P<0.001) **(Figure 1E)**.

The total effect of LLD/HC on the TMTB was β= 0.719 (P<0.001), the indirect effect of LLD/HC on the TMTB mediated by the SDMT was β= 0.381 (BootLLCI= 0.228，BootULCI=0.554). Furthermore, the remaining direct effect of LLD/HC on the TMTB was β= 0.339 (P=0.014), with the effect of LLD/HC on the SDMT being β= -0.706 (P<0.001) and SDMT on TMTB being β=-0.540 (P<0.001) **(Figure 1F)**.

The total effect of LLD/HC on the WMT was β= -0.885 (P<0.001), the indirect effect of LLD/HC on the WMT mediated by the SDMT was β=-0.245 (BootLLCI=-0.384，BootULCI=-0.122). Furthermore, the remaining direct effect of LLD/HC on the WMT was β=-0.640 (P<0.001), with the effect of LLD/HC on the SDMT being β= -0.695 (P<0.001) and SDMT on WMT being β=0.352 (P<0.001) **(Figure 1G)**.

**Comparison of the static fALFF, ReHo and FC of hippocampal subregions**

In analyses of static fALFF and Reho, there were no significant differences in hippocampal subregions between the LLD group and the HC group (**S.Figure 1)**.

In analyses of static FC of hippocampal subregions, differences between LLD and HC group were shown in **S.Figure 2**, (*P*<0.05,uncorrected). There was no significant difference when multiple comparisons (GRF, voxel *P*<0.001, cluster *P*<0.01)) was applied.

**S.Figure 1 Comparison of the static fALFF and ReHo of hippocampal subregions between the LLD group and the HC group** fALFF, fractional amplitude of low-frequency fluctuations; Reho, regional homogeneity. LLD, late-life depression; HC, healthy control; cHipp: caudal hippocampus. rHipp: rostral hippocampus. L, left; R, right.


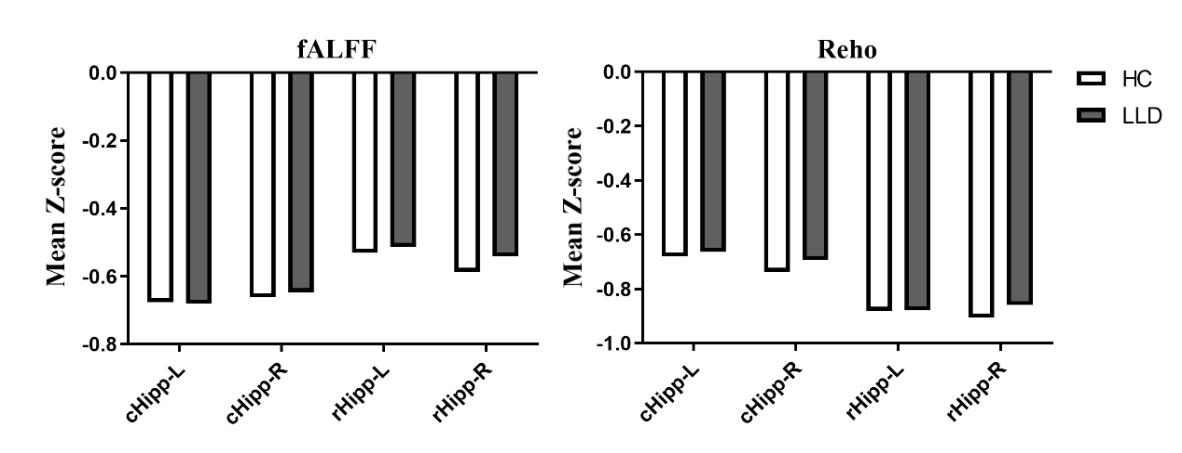


**S.Figure 2 Differences of FC between LLD and HC group (*P*<0.05,uncorrected) FC, functional connectivity; LLD, late-life depression; HC, healthy control;** L, left; R, right.


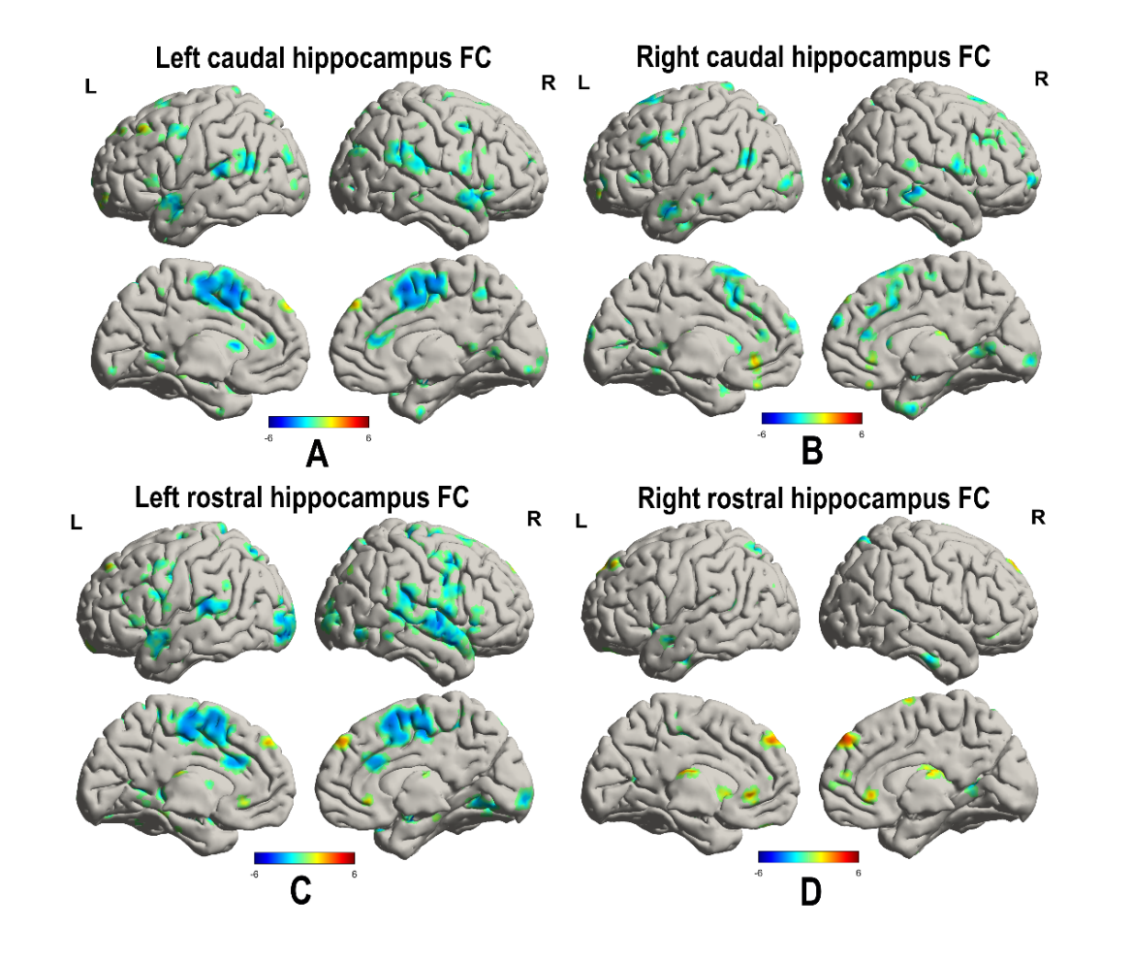


**Comparison of dfALFF, dReHo and dFC of hippocampal subregions with 30 and 70 TR as sliding window size**

In analyses of dfALFF, the LLD group presented lower dfALFF in the left rostral compared with the HC group when 70 TR was applied as sliding window size (F=5.98, P=0.015 **S.Figure 3B**), while no significant differences were observed with 30 TR (F=3.54, P=0.061 **S.Figure 3A**) or 50 TR (F=3.37,P=0.068 **Figure 3A**). There were no other significant differences in dfALFF of hippocampal subregions between the LLD group and the HC group.

In analyses of dReHo, the LLD group presented lower dReHo hippocampus in the left rostral compared with the HC group when 30 TR (F=5.52, P=0.020, **S.Figure 3C**), 50 TR (F=5.48, P=0.020 **Figure 3B**) and 70TR (F=5.96,P=0.015 **S.Figure 3D**) was applied as sliding window size. There were no other significant differences in other dReho of hippocampal subregions between the LLD group and the HC group.

In analyses of dFC with 30 TR as sliding window size, the LLD group exhibited decreased dFC between the left caudal hippocampus and right inferior frontal gyrus (opercular), left caudal hippocampus and right middle frontal gyrus (two clusters), left caudal hippocampus and right superior frontal gyrus (**S.Figure 4A, S.Table 1)**; right caudal hippocampus and right middle frontal gyrus (**S.Figure 4B, S.Table 1)**; left rostral hippocampus and right middle frontal gyrus (orbital) (**S.Figure 4C, S.Table 1)**; and the increased dFC between the right rostral hippocampus and left superior frontal gyrus (**S.Figure 4D, S.Table 1)**.

In analyses of dFC with 70 TR as sliding window size, the LLD group exhibited decreased dFC between the left caudal hippocampus and right middle frontal gyrus, left caudal hippocampus and right inferior frontal gyrus (triangular) (**S.Figure 5A, S.Table 2)**; right caudal hippocampus and right middle frontal gyrus (**S.Figure 5B, S.Table 2)**; left rostral hippocampus and right inferior frontal gyrus (orbital) (**S.Figure 5C, S.Table 2)**; and the increased dFC between the right rostral hippocampus and left superior frontal gyrus (**S.Figure 5D, S.Table 2)**.

**S.Figure 3 Comparison of the dfALFF and dReHo of hippocampal subregions between the LLD group and the HC group with 30 and 70 TR as sliding window size** dfALFF: dynamic dynamic fractional amplitude of low-frequency fluctuations; dReho, dynamic regional homogeneity. LLD, late-life depression; HC, healthy control; TR, Time of Repetition; cHipp: caudal hippocampus. rHipp: rostral hippocampus. L, left; R, right.


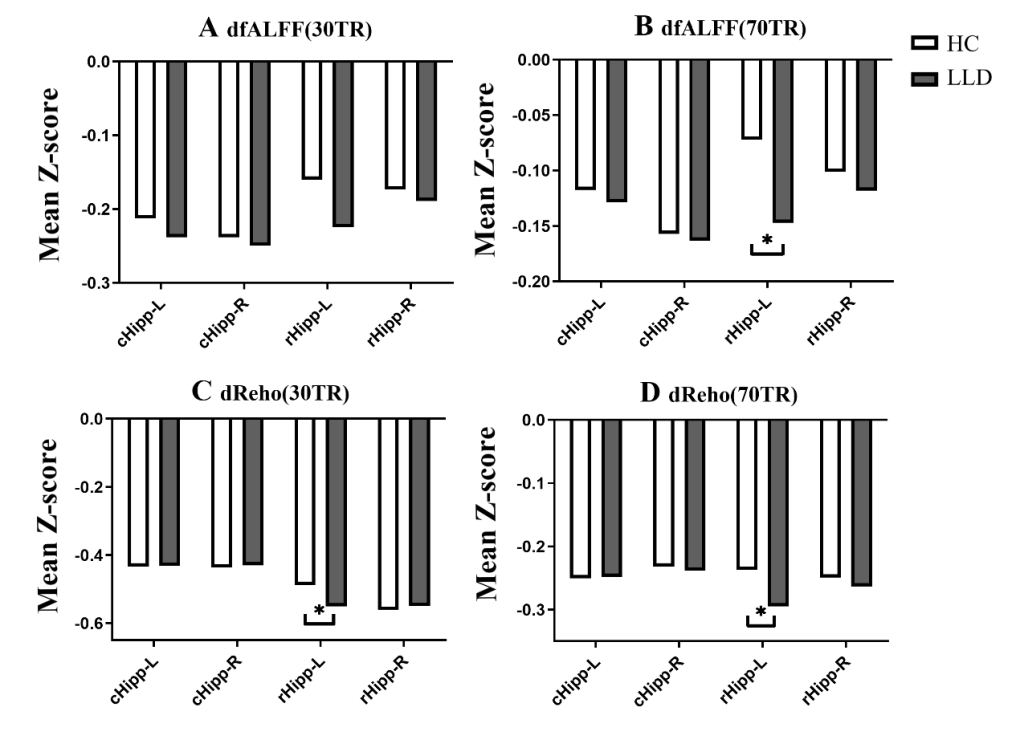


**S.Figure 4 Comparison of the dFC of hippocampal subregions between the LLD group and the HC group (30TR).** The LLD group exhibited decreased dFC between the **A.** left caudal hippocampus and right inferior frontal gyrus (opercular), left caudal hippocampus and right middle frontal gyrus (two clusters), left caudal hippocampus and right superior frontal gyrus; **B.** right caudal hippocampus and right middle frontal gyrus; **C.** left rostral hippocampus and right middle frontal gyrus (orbital); and **D.** the increased dFC between the right rostral hippocampus and left superior frontal gyrus. dFC: dynamic functional connectivity; LLD, late-life depression; HC, healthy control; TR, Time of Repetition; L, left; R, right.


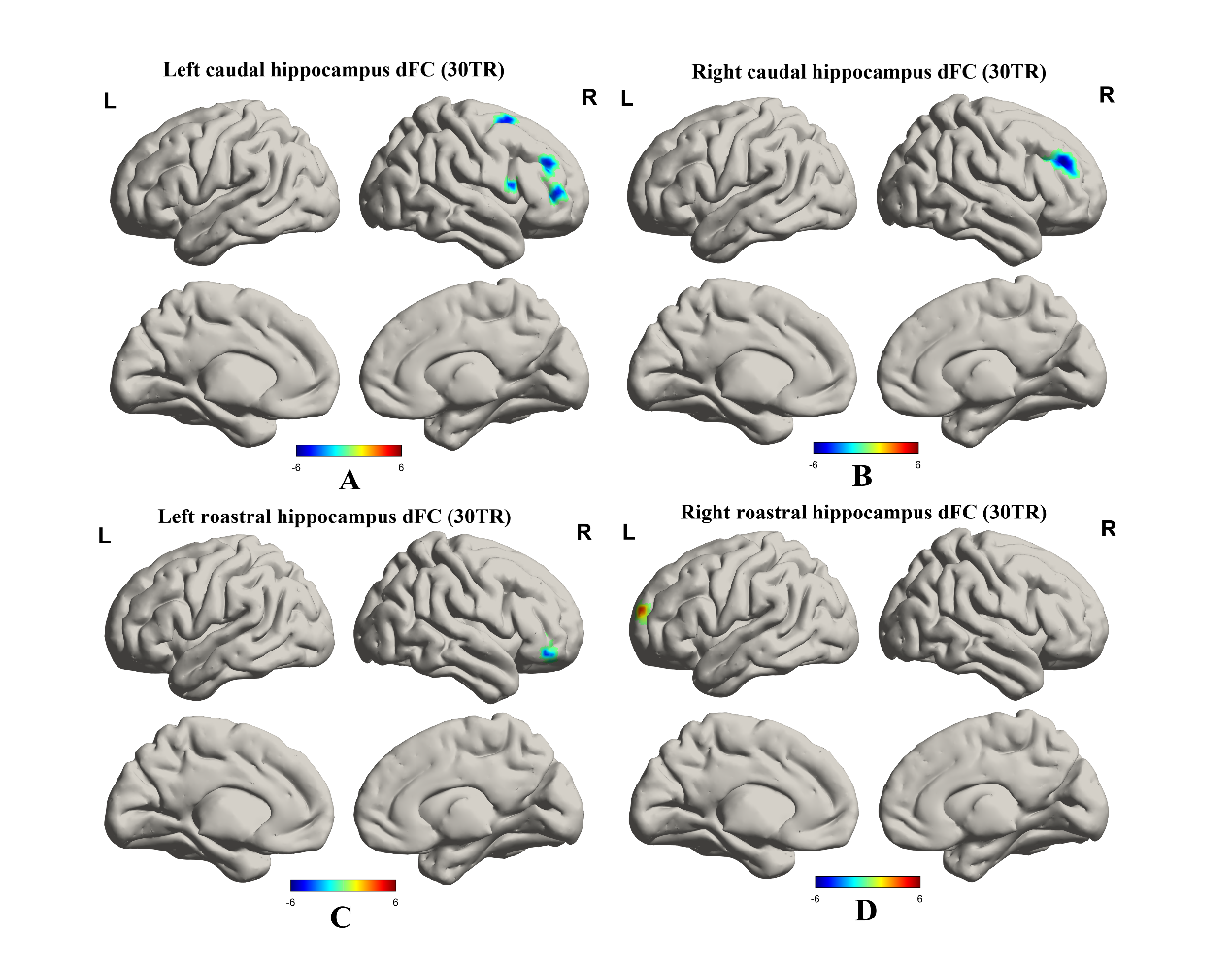


**S.Figure 5 Comparison of the dFC of hippocampal subregions between the LLD group and the HC group (70TR).** The LLD group exhibited decreased dFC between the **A.** left caudal hippocampus and right middle frontal gyrus, left caudal hippocampus and right inferior frontal gyrus (triangular); **B.** right caudal hippocampus and right middle frontal gyrus; **C.** left rostral hippocampus and right inferior frontal gyrus (orbital); and **D.** the increased dFC between the right rostral hippocampus and left superior frontal gyrus. dFC: dynamic functional connectivity; LLD, late-life depression; HC, healthy control; TR, Time of Repetition; L, left; R, right.


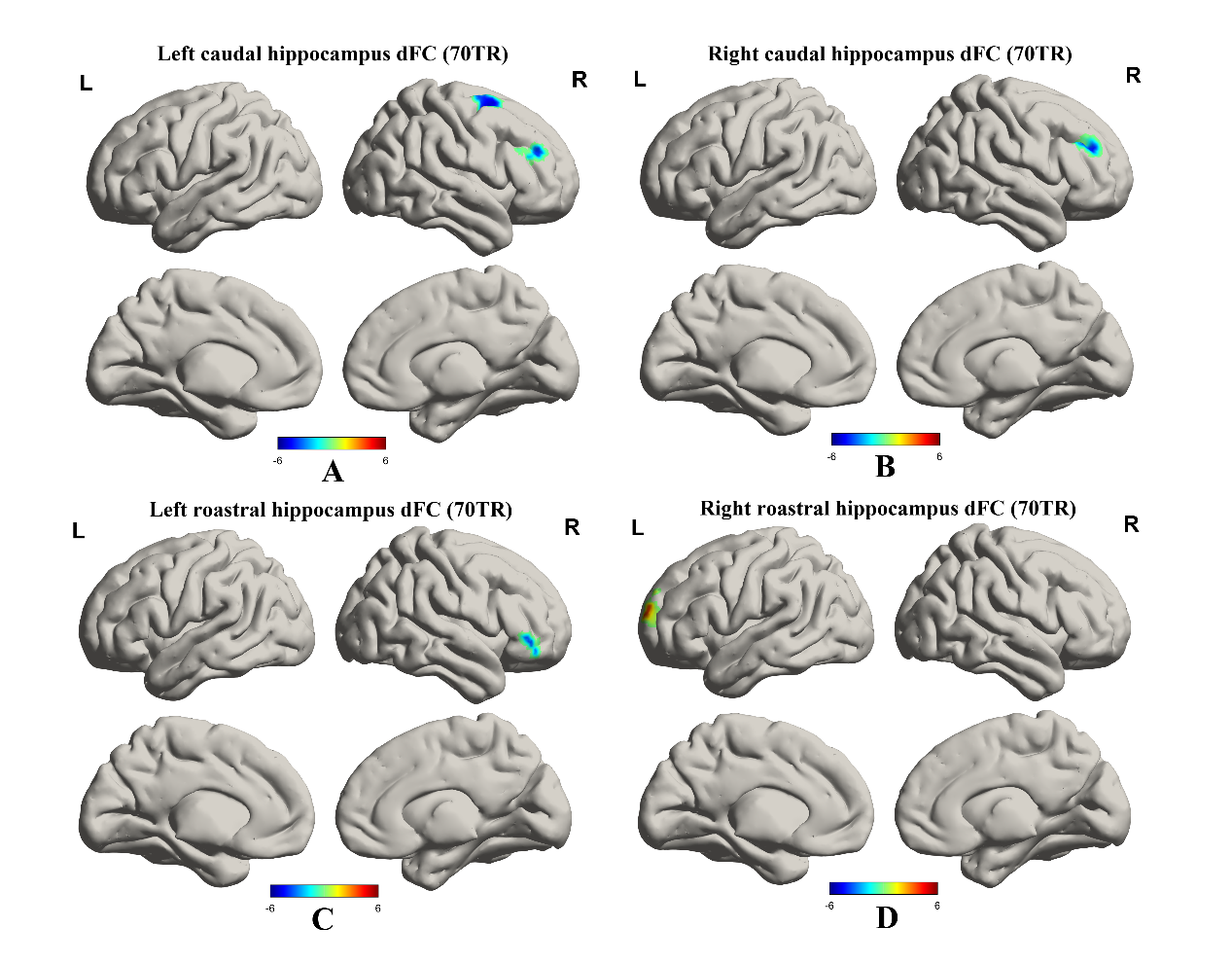


**S.Table 1** **Comparison of the dFC of hippocampal subregions between the LLD**

**group and the HC group (30TR)**. dFC: dynamic functional connectivity; LLD, late-life depression; HC, healthy control;TR, Time of Repetition MNI, Montreal Neurological Institute coordinates.

|  | Brain Regions | Peak MNI | | | | Cluster size | Peak  Intensity |
| --- | --- | --- | --- | --- | --- | --- | --- |
|  |  | x | y | | z |  |  |
| **Left caudal hippocampus dFC** | | | | | | | |
|  | Right inferior frontal gyrus (opercular)  Right middle frontal gyrus  Right middle frontal gyrus  Right superior frontal gyrus | 57  48  42  30 | 12  48  42  9 | 15  9  30  63 | | 21  26  36  31 | 4.46  4.57  4.58  4.61 |
| **Right caudal hippocampus dFC** | | | | | | | |
|  | Right middle frontal gyrus | 42 | 39 | 30 | | 116 | 5.93 |
| **Left rostral hippocampus dFC** | | | | | | | |
|  | Right middle frontal gyrus (orbital) | 33 | 45 | -9 | | 63 | 5.28 |
| **Right rostral hippocampus dFC** | | | | | | | |
|  | Left superior frontal gyrus | -18 | 54 | 12 | | 44 | 4.70 |

**S.Table 2 Comparison of the dFC of hippocampal subregions between the LLD**

**group and the HC group (70TR).** dFC: dynamic functional connectivity; LLD, late-life depression; HC, healthy control;TR, Time of Repetition; MNI, Montreal Neurological Institute coordinates.

|  | Brain Regions | Peak MNI | | | | Cluster size | Peak  Intensity |
| --- | --- | --- | --- | --- | --- | --- | --- |
|  |  | x | y | | z |  |  |
| **Left caudal hippocampus dFC** | | | | | | | |
|  | Right middle frontal gyrus  Right inferior frontal gyrus (triangular) | 33  45 | 3  33 | 57  24 | | 85  38 | 5.39  4.58 |
| **Right caudal hippocampus dFC** | | | | | | | |
|  | Right middle frontal gyrus | 42 | 42 | 27 | | 41 | 4.81 |
| **Left rostral hippocampus dFC** | | | | | | | |
|  | Right inferior frontal gyrus (orbital) | 39 | 45 | -3 | | 31 | 4.48 |
| **Right rostral hippocampus dFC** | | | | | | | |
|  | Left superior frontal gyrus | -21 | 54 | 12 | | 64 | 4.66 |
